# The Lombard effect in male ultrasonic frogs: Regulating antiphonal signal frequency and amplitude in noise

**DOI:** 10.1038/srep27103

**Published:** 2016-06-27

**Authors:** Jun-Xian Shen, Zhi-Min Xu

**Affiliations:** 1 State Key Laboratory of Brain and Cognitive Science, Institute of Biophysics, Chinese Academy of Sciences, Beijing, China

## Abstract

Acoustic communication in noisy environments presents a significant challenge for vocal animals because noise can interfere with animal acoustic signals by decreasing signal-to-noise ratios and masking signals. Birds and mammals increase call intensity or frequency as noise levels increase, but it is unclear to what extend this behavior is shared by frogs. Concave-eared torrent frogs (*Odorrana tormota*) have evolved the capacity to produce various calls containing ultrasonic harmonics and to communicate beside noisy streams. However, it is largely unclear how frogs regulate vocalization in response to increasing noise levels. We exposed male frogs to various levels of noise with playback of conspecific female courtship calls and recorded antiphonal signals and spontaneous short calls. Males were capable of rapidly adjusting fundamental frequency and amplitude of antiphonal signals as noise levels increased. The increment in fundamental frequency and amplitude was approximately 0.5 kHz and 3 dB with every 10 dB increase in noise level, indicating the presence of noise-dependent signal characteristics. Males showed the noise-tolerant adaption in response to female calls in noise level from 40 to 90 dB SPL. The results suggest that the noise-dependent signal characteristics in *O. tormota* have evolved as a strategy to cope with varying torrent noise.

The Chinese concave-eared torrent frogs (*Odorrana tormota*, Ranidae)^1^ inhabit vegetation along mountain streams at elevations of approximately 150–700 m, where the ambient noise generated by running water is broadband (about 0.1–48 kHz) with a peak near 1.7 kHz. During breeding season at male’s calling sites, the background noise intensity is usually between 80 and 95 dB SPL (sound pressure level, 0 dB re. 20 *μ*Pa RMS). Males^3^,^4^,^5^ and females^6^ produce high-pitched calls containing spectral energy in the ultrasonic range (above 20 kHz), and their hearing extends to approximately 35 kHz for males^5^ and 16 kHz for females^7^. This exceeds the upper limits of most anurans’ frequency sensitivity, e.g., 8.2 kHz, as determined by the auditory sensitivity of nerve fibres innervating the basilar papilla^9^, or 5 kHz, as supported by current psychoacoustical data^10^. However, it is largely unclear whether or not concave-eared frogs exhibit noise-dependent vocal characteristics as a short-term response. An animal increases its vocal amplitude and changes other acoustic parameters in response to an increase in background noise, called the Lombard effect^11^,^12^,^13^ and found in mammals^14^,^15^,^16^,^17^,^18^,^19^ and some birds^20^,^21^,^22^,^23^,^24^,^25^. It is clear that some species exhibit the Lombard effect^26^, some don’t^27^, and that it is unclear how widespread the phenomenon is in frogs.

However, some frogs living in habitat of high levels of stream noise produce vocalizations containing remarkable frequency modulations and/or high frequencies beyond the noise spectral range to overcome noise interference^3^,^2^8–3^1^. Males of the Puerto Rican treefrog *Eleutherodactylus coqui* cease responding to synthesized calls if they are accompanied by intense broadband noise^32^. Males of the frog *Eupsophus calcaratus* increased their call rate and duration in response to exposures of band-pass noise between 700 and 2,700 Hz of various intensities (66–78 dB SPL RMS)^33^. Green frogs increase the peak frequency of their calls when suddenly exposed to loud anthropogenic noise^34^. Playback treatments caused spring peepers *Pseudacris crucifer* to produce shorter calls, the high-frequency noise treatment caused them to lower the frequency of their calls immediately after the noise ceased, and call rate did not change in response to playback^35^. Males of Cope’s grey treefrog *Hyla chrysoscelis* did not regulate call amplitude, although increasing noise levels ranging between 40 dB and 70 dB induced predictable changes in call duration and rate^27^. The results from the grey treefrog do not support the hypothesis that voice amplitude regulation is a generic vertebrate mechanism for coping with noise^27^. A recent research demonstrates that male túngara frogs (*Physalaemus pustulosus*) exhibit the Lombard effect, increasing call amplitude linearly with noise level in response to low-frequency noise (overlapping the species’ call range) but not to high-frequency (nonoverlapping) noise^26^.

Our study subject, *Odorrana tormota*, is a nocturnal ranid species living beside noisy streams in southern China^1^. Males of this species are unique and their eardrums are deeply sunken at the far end of “ear canals” with unobstructed openings at the body surface^3^. Males produce ultrasonic calls, preventing frequency overlap with stream noise, as long-term acoustic adaptations^5^,^6^,^7^. Recently, an efficient form of courtship in the frog was reported, in which males answer the female courtship calls (FC) with short-latency antiphonal calls with different fundamental frequencies (*F*_*0*_) in the field and indoors, that are positively correlated with the level of ambient noise, and receptive males exhibited hyperacute phonotaxis toward FC playbacks^6^. Antiphonal vocalization has been regarded to have an important function in nocturnal or subterranean animals living in an environment where visual cues are unreliable^36^. In this study, we recorded and compared antiphonal signals (ASs, elicited with FC playbacks) and spontaneous short calls (SSCs, in the absence of FC playback, obtained in respective noise) in response to increasing noise levels to investigate whether or not and how males of *O. tormota* cope with various levels of noise exposures.

## Results

A total of 114 receptive males of *O. tormota* were tested for each condition in which broadcasting randomly with combined FC noise at different SNR of 32, 22, 12, and 2 dB, respectively, and a total of 12 FC playbacks for each trial (see METHODS). The antiphonal signals (ASs, elicited with FC playbacks) and spontaneous short calls (SSCs, in the absence of FC playback, obtained in noise) were recorded. The AS number per frog was from 0 up to 8 (median = 2.36) dependent on SNR, but only one AS per frog was collected for analysis at each SNR condition. The total number of ASs was 269, i.e., 36, 100, 93, and 40 ASs at the SNR of 32, 22, 12 and 2 dB respectively.

### Natural torrent noise

**Figure 1:**
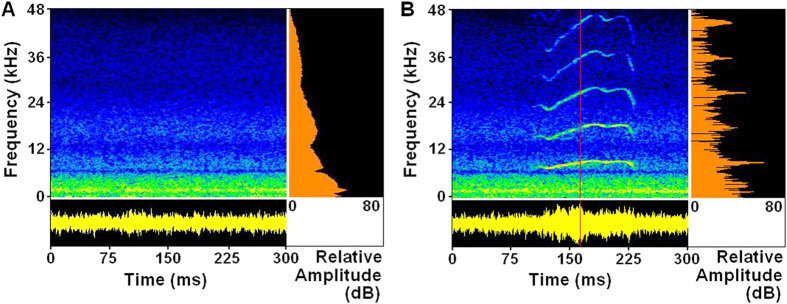
Natural torrent noise and combined FC noise, illustrating the acoustic features. (**A**) A clip of natural torrent noise at 83 dB SPL, top left panel: spectrogram; bottom panel: amplitude envelop; right panel: amplitude spectrum. (**B**) A clip of the combined FC noise with the SNR of +2 dB (FC playback at 85 dB SPL, noise at 83 dB SPL), right panel: instantaneous amplitude spectrum, taken at indicated point of the red vertical cursor. Data were saved as wave files and analyzed (FFT, 1024), and displayed using SELENA, a custom-designed program (S. Andrzheevski, St. Petersburg)^4^. Ordinate for top left and right panel: frequency (kHz), ranging from 0 to 48 kHz; abscissa for right panel: relative amplitude (dB), ranging from 0 to 80 dB; temperature range during recording: 17–18 °C.

The natural torrent noise was recorded at the upper reaches of the Taohua creek (elevations 650–700 m). Figure 1A shows the spectral characteristics and amplitude envelop of the noise at 83 dB SPL (0 dB = 20 *μ*Pa RMS). The noise contains prominent acoustic energy in low-frequency range with a peak near 1.7 kHz, decaying by 5 dB at 100 Hz, by 20 dB at 8.0 kHz, by 37 dB at 20 kHz, and by 57 dB at 48 kHz. The spectrum does not change with the average noise level. Figure 1B illustrates a clip of the combined FC noise with the signal-to-noise ratio (SNR) of +2 dB, depicting 5 harmonics of the FC (duration 140 ms) with a fundamental frequency (*F*_*0*_) at about 9.0 kHz. Apparently, the natural noise overlaps partly with FC’s harmonics only in a low-frequency range (0.1–20.0 kHz).

### Two basic types of antiphonal signals

**Figure 2:**
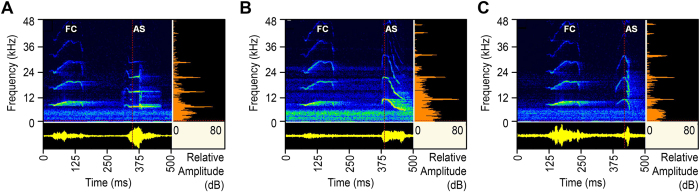
Two basic types of antiphonal signals elicited from male *O. tormota* with combined FC noise exposures. (**A**) A representative AS of CF-type, an unmodulated-frequency *F*_*0*_ of 6.9 kHz, at the SNR of 22 dB and noise level of 63 dB SPL. (**B**) A representative AS of FM-type, a mean *F*_*0*_ of 7.8 kHz, at the SNR of 12 dB and noise level of 73 dB SPL. (**C**) A representative AS of FM-type, upward and downward FM sweep, a mean *F*_*0*_ of 9.4 kHz, at the SNR of 22 dB and noise level of 63 dB SPL. Other data same as Fig. 1.

We recorded antiphonal signals (ASs) (elicited with FC playbacks) and spontaneous short calls (SSCs) (in the absence of FC playback, obtained in noise) from individual receptive males by using various combined FC noise exposures with 4 different SNRs (32, 22, 12, and 2 dB, respectively; respective noise level: 53, 63, 73, and 83 dB SPL; FC playback at 85 dB SPL). The ASs were single-note short calls (duration <100 ms; delay times or latency <400 ms, measured from the onset of FC playback) of two types, a constant-frequency tone (CF-type) (63.2%, 72 of 114 males) with an unmodulated-frequency *F*_*0*_ (Fig. 2A; *F*_*0*_ 6.9 kHz) and a frequency-modulated sweep (FM-type) (36.8%, 42 males). Most of the FM-type ASs were a downward sweep from 10.2 kHz to 5.3 kHz (Fig. 2B; mean of *F*_*0*_ 7.8 kHz) (78.6%, 33 of 42 males), and others were an upward and downward sweep between 6.5 kHz and 10.4 kHz (Fig. 2C; mean of *F*_*0*_ 9.4 kHz) (21.4%, 9 males).

### Noise-dependent fundamental frequency regulation of antiphonal signals

**Figure 3:**
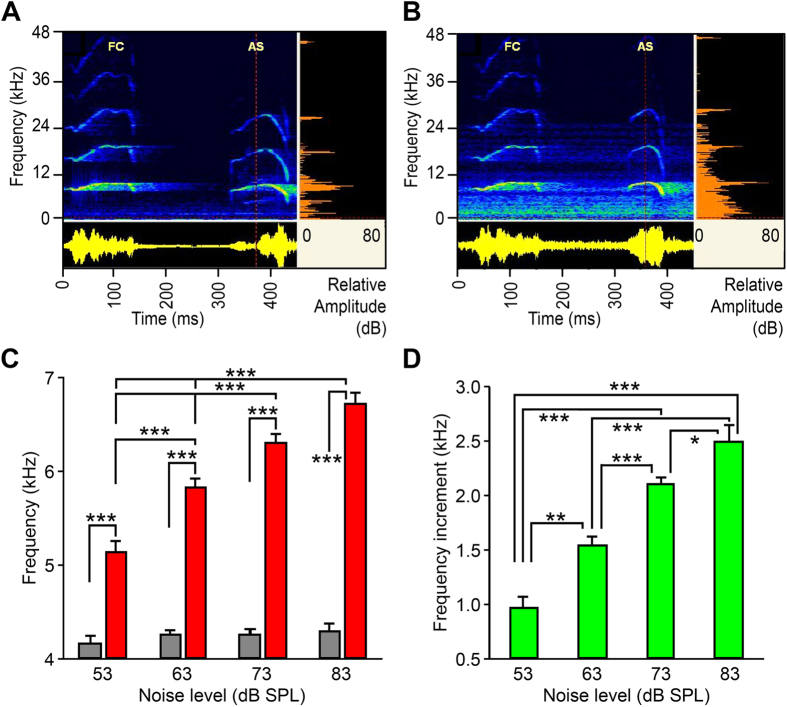
Noise-dependent antiphonal signals of approximate CF-type in *O. tormota*. (**A**) One male emitted an AS of high *F*_*0*_(8.0 kHz), longer duration (106 ms) and high amplitude (86 dB SPL) elicited by combined FC noise with SNR of 32 dB at noise level of 53 dB SPL. (**B**) The same male emitted an AS of higher *F*_*0*_(8.7 kHz), shorter duration (66 ms) and higher amplitude (89 dB SPL) elicited by combined FC noise with SNR of 12 dB at noise level of 73 dB SPL. (**C**) Averaged *F*_*0*_of ASs (red) increased significantly with increasing noise levels. SSCs (gray) show no significant difference in *F*_*0*_between various noise levels. (**D**) The *F*_*0*_ increments of ASs (relative to SSCs) increased significantly with increasing noise levels. The results in (**C**,**D**) are means + s.e.m. ***P < 0.001; **P < 0.01; *P < 0.05. Other data same as Fig. 1.

The effects of combined FC noise with different SNRs on antiphonal signals were evident. For the same male, when the SNR of combined FC noise decreased from 32 dB to 12 dB (i.e., respective noise level increased from 53 to 73 dB SPL), the average *F*_*0*_ of an approximate CF-type AS rose from 8.0 kHz (Fig. 3A) to 8.7 kHz (Fig. 3B), the duration shortened from 106 to 66 ms and the sound level increased from 86 to 89 dB SPL. As for all CF-type ASs recorded from 72 males, the population mean of *F*_*0*_of CF-type ASs exhibited statistically significant differences and positively correlated with increasing noise levels as determined by one-way ANOVA (Fig. 3C; *F*_3, 176_ = 29.093, *P* < 0.0001, *n* = 27, 66, 60, and 27 males, respectively, for noise level group of 53, 63, 73, and 83 dB SPL) with a dynamic range of *F*_*0*_from 5.14 to 6.72 kHz when noise level changed in a range from 53 to 83 dB SPL. A Tukey HSD post-hoc test revealed that there were statistically significant differences in the *f*_0_ of CF-type AS among the 53 dB SPL group (5136.1 ± 130.2 Hz), the 63 dB SPL group (5830.0 ± 89.8 Hz) (*P* < 0.0001), the 73 dB SPL group (6305.0 ± 83.2 Hz) (*P* < 0.0001), and the 83 dB SPL group (6716 ± 133.5 Hz) (*P* < 0.0001) (Fig. 3C). There were no statistically significant differences in the *f*_0_ of CF-type AS between the 73 dB SPL group and the 83 dB SPL group (*P* = 0.052). Under the same noise level, the mean *F*_*0*_ of ASs was significantly greater than that of SSCs (*P* < 0.0001), while the *F*_*0*_of SSCs did not differ among noise treatments (*F*_3, 170_ = 0.618, *P* = 0.604).

The *F*_*0*_ increments of both ASs versus SSCs rose significantly from 0.96 to 2.49 kHz with the noise level increasing, an average of 0.5 kHz with every 10 dB increase in noise level (Fig. 3D; *F*_3, 176_ = 30.354, *P* < 0.0001; *P* = 0.034 between the 73 dB SPL group and the 83 dB SPL group). The data demonstrate that the *F*_*0*_ variation of CF-type ASs was a function of the ambient noise level and males could regulate fundamental frequency of antiphonal signals in direct proportion to the level of noise exposure.

**Figure 4:**
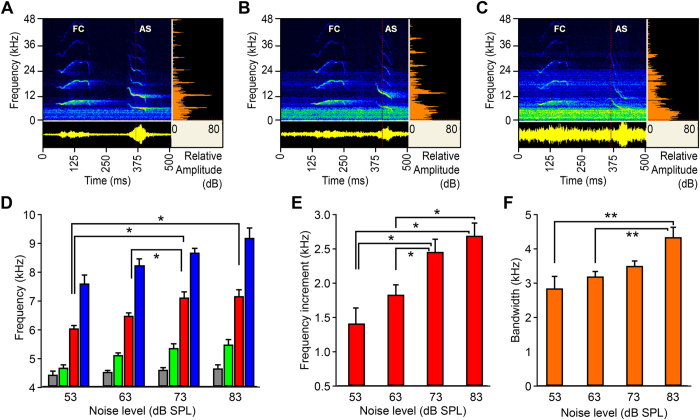
Noise-dependent antiphonal signals of an FM-type in male *O. tormota* elicited by combined FC noise exposures with various SNRs. (**A**) One male emitted an FM-type AS with the average *F*_*0*_ of 6.0 kHz at noise level of 63 dB SPL (SNR = 22 dB). (**B**) The same male emitted an FM-type AS with the average *F*_*0*_ of 6.3 kHz at noise level of 73 dB SPL (SNR = 12 dB). (**C**) The same male emitted an FM-type AS with the average *F*_*0*_of 7.4 kHz at noise level of 83 dB SPL (SNR = 2 dB). (**D**) Histogram illustrating the frequency and noise level relationship; maximum (blue), average (red) and minimum (green) of *F*_*0*_ of FM-type ASs and *F*_*0*_of SSCs (gray). (**E**) Histogram illustrating the increment of average *F*_*0*_ of FM-type ASs increased significantly with increasing noise levels. (**F**) Histogram illustrating the bandwidth of *F*_*0*_ of FM-type ASs increased significantly as increasing noise levels. The results in (**D–F**) are means + s.e.m. ***P < 0.001; **P < 0.01; *P < 0.05. Other data same as Fig. 1.

The FM-type ASs elicited from males by combined FC noise exposures with various SNRs also exhibited noise-dependent signal characteristics in the average and maximum of *F*_*0*_. As an example shown in Fig. 4A–C, when noise level increased from 63 dB SPL to 73, and 83 dB SPL, the corresponding maximum of *F*_*0*_ of ASs increased from 7.5 kHz (Fig. 4A), to 8.0 kHz (Fig. 4B) and 10.3 kHz (Fig. 4C), respectively. Similarly, the average *F*_*0*_ increased from 6.0 kHz to 6.3, and 7.4 kHz, respectively, and the bandwidth of FM sweeps became broader, increasing from 2.1 kHz to 2.7, and 5.1 kHz, respectively, whereas the minimum remained nearly unchanged between 5.2 and 5.4 kHz. Statistical analyses of all FM-type ASs recorded from 42 males show that both the population average and maximum *F*_*0*_ exhibited significant differences and positively correlated from 6.06 to 7.13 kHz for the average and from 7.70 to 9.15 kHz for the maximum, respectively, as noise levels increased (Fig. 4D) (*F*_3, 84_ = 5.418, *P* = 0.002 for average; *F*_3, 84_ = 3.587, *P* = 0.017 for maximum; *n* = 8, 34, 33, and 13 males, respectively, for noise level group of 53, 63, 73, and 83 dB SPL). A Tukey HSD post-hoc test revealed that there were statistically significant differences in average *f*_0_ of FM-type AS among the 53 dB SPL group (5988.6 ± 120.0 Hz), the 73 dB SPL group (7078.7 ± 198.4 Hz) (*P* = 0.016), and the 83 dB SPL group (7130 ± 223.6 Hz) (*P* = 0.032), and between the 63 dB SPL group (6433.7 ± 131.5 Hz) and the 73 dB SPL group (7078.7 ± 198.4 Hz) (*P* = 0.024) (Fig. 4D). There were no statistically significant differences between the 53 dB SPL group and the 63 dB SPL group (*P* = 0.533), and among the 63 dB SPL group, the 73 dB SPL group and the 83 dB SPL group (*P* = 0.157).

The averages of *F*_*0*_ were significantly different from those of SSCs at respective noise levels (*P* < 0.0001). However, the minimum *F*_*0*_ did not change statistically from 4.66 to 5.5 kHz with noise treatments (*F*_3, 84_ = 2.638, *P* = 0.055). Furthermore, the increment of average *F*_*0*_ of FM-type ASs relative to SSCs increased significantly from approximately 1.39 to 2.67 kHz in the noise level range from 53 to 83 dB SPL (Fig. 4E; *F*_3, 84_ = 5.518, *P* = 0.002), having an average increment of 0.44 kHz per 10 dB of noise level increase. Figure 4F illustrates the relationship between the bandwidth of FM-type ASs and noise levels, indicating the bandwidth presented a marked increase from 2.83 to 4.33 kHz with increasing noise levels (*F*_3, 84_ = 5.262, *P* = 0.002). It is evident that the both the *F*_*0*_ average and maximum of FM-type ASs were also regulated depended on the level of noise exposure.

### Noise-dependent amplitude regulation of antiphonal signals

**Figure 5:**
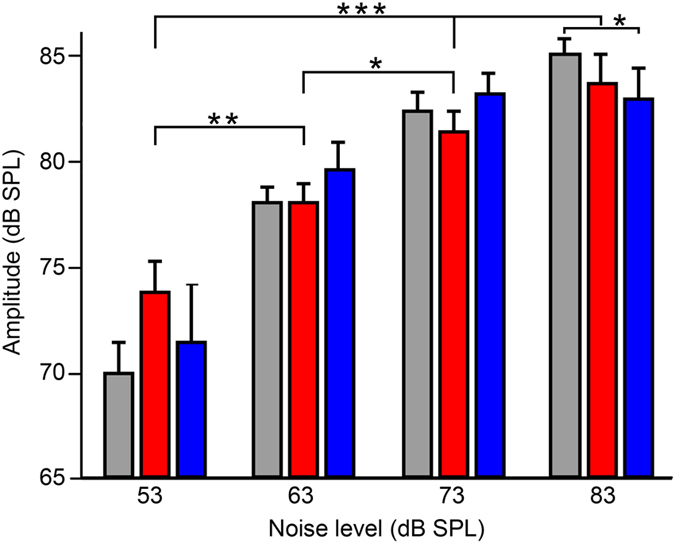
The Lombard effect occurred in males of *O. tormota*. The amplitudes of both ASs and SSCs increased significantly as the level of noise exposure increased from 53 to 83 dB SPL (red: *n* = 22, 66, 51, and 11 males, respectively, for noise level group of 53, 63, 73, and 83 dB SPL for CF-type ASs; blue: *n* = 4, 33, 24, and 8 males, respectively, for noise level group of 53, 63, 73, and 83 dB SPL for FM-type ASs; gray: *n* = 41, 41, 32, and 5 males, respectively, for noise level group of 53, 63, 73, and 83 dB SPL for SSCs; ***P < 0.001; **P < 0.01; *P < 0.05). The results are means + s.e.m. Error bars for the panel indicate s.e.m.

To clarify whether or not the Lombard effect occurs in frogs, we analyzed the amplitudes of males’ ASs elicited by combined FC noise exposures with various SNRs. We found that males increased amplitude of ASs in response to increasing noise level significantly and positively correlated with noise level, on average, with an amplitude increment of 3 dB with every 10 dB increase in noise level as determined by one-way ANOVA (*F*_3, 146_ = 11.13, *P* < 0.0001, *n* = 22, 66, 51, and 11 males, respectively, for noise level group of 53, 63, 73, and 83 dB SPL for CF-type ASs; *F*_3, 65_ = 6.405, *P* < 0.001, *n* = 4, 33, 24, and 8 males, respectively, for noise level group of 53, 63, 73, and 83 dB SPL for FM-type ASs) (Fig. 5). A Tukey HSD post-hoc test revealed that there were statistically significant differences in amplitude of CF-type AS among the 53 dB SPL group (62.7 ± 1.3 dB), the 63 dB SPL group (68.0 ± 0.9 dB) (*P* = 0.005), the 73 dB SPL group (71.3 ± 0.7 dB) (*P* = 0.0001), and the 83 dB SPL group (72.6 ± 1.6 dB) (*P* = 0.0001), and between the 63 dB SPL group and the 73 dB SPL group (*P* = 0.029) (Fig. 5). There were statistically significant differences in amplitude of FM-type AS among the 53 dB SPL group (61.4 ± 4.5 dB), the 63 dB SPL group (69.5 ± 1.1 dB) (*P* = 0.032), the 73 dB SPL group (73.1 ± 0.7 dB) (*P* = 0.001), and the 83 dB SPL group (72.8 ± 0.8 dB) (*P* = 0.006) (not shown in Fig. 5). In fact, increases in noise level resulted in significant rises in the amplitude of SSCs (in the absence of FC playbacks) as well (*F*_3, 115_ = 28.35, *P* < 0.0001, *n* = 41, 41, 32, and 5 males, respectively, for noise level group of 53, 63, 73, and 83 dB SPL for SSCs). Under the same level of noise, there were no significant differences in amplitude between ASs and SSCs (*P* > 0.11), except the comparison between FM-type ASs and SSCs at 83 dB SPL, in which mean amplitude of FM-type ASs was lower than that of SSCs (*P* = 0.02) (Fig. 5). The minimum signal-to-noise ratios of the recorded AS and SSC were −8.3 dB and 1.5 dB, respectively. The findings indicate that males of *O. tormota* emit signals of higher intensity as noise levels increase, revealing the presence of the Lombard effect in the frog.

Furthermore, the duration of all CF-type and FM-type ASs at each SNR condition was analyzed. For SNR of 32 dB, the average duration was 56.6 ± 15.5 ms (mean ± SD; *n* = 36); 59.2 ± 20.7 ms (*n* = 100) for SNR of 22 dB, 59.7 ± 21.2 ms (*n* = 93) for SNR of 12 dB, and 60.2 ± 19.5 ms (*n* = 40) for SNR of 2 dB, respectively, showing a little longer about 3 ms with increasing noise levels with the exception of data for a single CF-type AS (shown in Fig. 3). Statistically, the population mean of AS duration was 59.2 ± 20.1 ms and there was no significant difference in the AS duration between each SNR condition (ANOVA, *F*_3, 265_ = 0.24, *P* = 0.868).

The delay times of ASs measured from the FC onset at each SNR condition was analyzed as follows: 283.0 ± 40.8 ms for SNR of 32 dB (*n* = 36), 273.8 ± 44.0 ms for SNR of 22 dB (*n* = 100), 272.0 ± 38.1 ms for SNR of 12 dB (*n* = 93), and 278.2 ± 43.7 ms for SNR of 2 dB (*n* = 40). When SNR was reduced from 32 dB to 22 or 12 dB, the AS delay times became shorter of 10 ms, whereas there was a delay of about 5 ms for SNR of 2 dB. Statistically, there was no significant difference in AS delay times between each SNR condition (ANOVA, *F*_3, 265_ = 0.700, *P* = 0.553).

## Discussion

Antiphony enables to acknowledge that one’s signal was received with certainty^36^. Antiphonal signals (ASs) evoked from males due to FC stimulation play a vital role in courtship for mate attraction, particularly for nocturnal frogs^6^. Thus, it is of significance to know the influence of noise levels on ASs and how males adjust ASs to ambient noise. The present data first demonstrate that males can simultaneously regulate noise-dependent frequency and amplitude of ASs. First, the increases in noise level result in a significant increase in the fundamental frequencies (*F*_*0*_) of ASs, about 0.5 kHz with every 10 dB increase in noise level (Figs 3D and 4E). This frequency shift may provide a masking release of 1–3 dB, a case seldom found in other anuran amphibians^13^,^2^7,^37^. Second, the amplitudes of both ASs and SSCs were positively correlated with noise level (see Fig. 5), about 3 dB with every 10 dB increase in noise level. These findings reveal the presence of the Lombard effect in the concave-eared frogs. This dynamic response of males may reduce noise masking of their response to females. Third, average population ASs duration of around 60 ms was little affected by noise in *O. tormota*, with the exception of data for a single CF-type AS (shown in Fig. 3). By contrast, increases in noise level resulted in significant increases in call duration in Cope’s grey treefrog^27^ and in leptodactylid frogs of the temperate austral forest^33^,^3^8; in both cases, call amplitude did not change, though a little support for noise-dependent voice amplitude regulation was found in leptodactylid frogs. However, male túngara frogs exhibited a Lombard effect in the range of 1–3 dB with every 10 dB increase in noise, showing call amplitude linearly with low-frequency noise level, but no frequency shift in the frog^26^.

Next, we usually recorded ASs from receptive males when the noise levels were between quiet (approximately 40 dB SPL) indoors and noisy conditions (up to 90 dB SPL) in field^6^. It suggests that the noise-tolerant adaptations in response to female calls have developed in the auditory and vocal pathways of *O. tormota*. We found that various levels of natural noise exposures (ranging from 35 to 85 dB SPL) have little effect on hearing in males^39^, implying the males are noise-tolerant in the noise level range. In addition, AS was seldom elicited from males when the signal to noise ratio (SNR) was set to less than −5 dB.

To summarize, we have shown that male concave-eared frogs have evolved the capacity to regulate both frequency and amplitude of ASs to answer female courtship calls under different noise levels as a strategy to deal with varying torrent noise. Nevertheless, these findings are dissimilar to those in echolocation bats^19^ and in urban birds^40^, in which call amplitude and frequency shifts occurred independent of one another. The presence of the Lombard effect in male concave-eared frogs (they change both frequency and amplitude of ASs in different levels of noise exposure; For SSC, only amplitude is modified, not frequency) and túngara frogs (they change call amplitude, not frequency, in response to noise)^26^ shows that there are at least two different mechanisms to deal with noise, as suggested by Slabbekoorn *et al*.^40^. It could support the speculation that the Lombard effect may be ‘a synapomorphy of all amniotes,’ not only ‘the outcome of a convergent evolution in birds and mammals’^13^. Noise acts as a strong selective force in sculpting acoustic communication systems, though the essential neuronal mechanisms mediating noise-dependent signal characteristics are less known.

## Methods

### Ethics statement

The playback experiments were carried out under the approval of the Animal Care and Use Committee of the Institute of Biophysics, Chinese Academy of Sciences in an accredited experimental room (GL 9106). All sampling procedures and experimental manipulations were specifically approved as part of obtaining the field permit. All experiments were performed in accordance with the approved guidelines.

### Frogs

Five *O. tormota* males were captured each night along the Taohua Creek (30°06′ N, 118°10′ E) in the Hot Spring area of the Huangshan Scenic Zone, maintained by Forestry Department of Anhui Province, which granted permission to JXS for conducting scientific research in the area (Forestry Department of Anhui Province, No. WLP 2011–1) from April 11 to May 5 in 2011–2012. The males were kept individually in multihole plastic baskets (30-cm upper diameter, 20-cm bottom diameter, 40-cm height) for acoustic playback experiments. A total of 114 receptive males were used in the present study from 1900 to 2200 h. After audio recording, unharmed males were released within 24 h of capture at the locations about 50 m away from their respective capture sites to ensure that each male was only tested once. The temperature and humidity ranges indoors during the sound recordings were 17–18 °C and 50–70%, respectively.

### Acoustic stimuli

Natural torrent noise along the upper reaches of the creek (elevations 650–700 m) was recorded using a high-resolution digital recorder (Type 722, Sound Devices, USA; frequency range 10 Hz–96 kHz), a 1/4-inch pre-polarized condenser microphone (Type 40 BE, frequency response 4 Hz–100 kHz, +/− 3 dB, G.R.A.S., Denmark) and a preamplifier (Type 26 CB, G.R.A.S., Denmark) positioned at 45° from the water surface, at a distance of 3 m from the noise source and a height of 1.5 m. This is at a typical location of a calling male. A 1,000 Hz tone at 94 dB SPL produced by a sound level calibrator (Type 4231, Brüel & Kjaer, Denmark) was used as a reference signal. Compared with the reference signal, the sound pressure levels of various natural noise exposures were adjusted to 4 different levels ranging from 53 to 83 dB SPL in steps of 10 dB (time window 140 ms, the same duration as the signal FC length) by the Praat software package for the analysis of phonetics (from University of Amsterdam, The Netherlands, and maintained by Paul Boersma and David Weenink)^40^, and the spectrum of the noise exposure does not change with the average noise level. Then, acoustic stimuli consisting of the female call playbacks (FC, duration 140 ms at the level of 85 dB SPL)^6^ combined with 4 different levels of natural noise (noise duration of 12 s; FC playback started 9 s after noise onset), here after referred to as “FC noise” with different SNR of 32, 22, 12, and 2 dB, respectively. The noise did not have a short ramp-on and off.

### Acoustic playback experiments

Experiments were carried out from 1930 to 2200 h in a quiet and darkened indoor room with background noise at 40 dB SPL, ~1 km from the frog’s natural habitat. Combined FC noise with 4 different SNRs was broadcast through a power amplifier and a full range speaker unit (FE87E, reproduction frequency response: 140 Hz to 30 kHz, 3 inch, 8 Ω, Fostex, Japan) with 3-s intervals of silence at the rate of 1 FC per 15 s over a 3-min period, a total of 12 FC playbacks for each trial, and 3-min silent periods between trials. A total of two 3-min trials per individual male were conducted for each condition. During recording sessions, the speaker unit was positioned 1 m away from a captive male frog, which remained motionless on the side of plastic basket covered with gauze. The same microphone and preamplifier were mounted on a tripod and positioned about 45° from the ground surface and 10 cm from the frog to monitor its vocalizations. In the experiments, 4 different FC noises were randomly applied, and antiphonal signals (ASs, elicited from males with FC playbacks) and spontaneous short calls (SSCs, without FC playbacks) were recorded using the same recorder (a sampling rate of 96 kHz). The main parameters for ASs and SSCs, e.g., basic types of calls, sound spectrograms, peak-to-peak amplitude (mean, maximum, minimum in dB SPL), duration and pitch (*F*_*0*_, maximum, minimum, average and sweep bandwidth), were measured with the Praat software package^41^ and displayed using SELENA, a custom-designed program (S. Andrzheevski, St. Petersburg)^4^,^4^2. The sound pressure levels of respective ASs and SSCs buried in various levels of noise were determined based on the decibel calculation of combining sound sources^43^,^4^4. Frogs did not call during the 3 sec silent intervals.

### Statistics

To analyze the effect of the exposure to natural noise, we measure and compared the main parameters of ASs elicited with FC playbacks presented at the same 85 dB SPL and SSC from the males of *O. tormota* at 4 different SNR conditions. The AS number per trial per male was dependent on a given SNR in the range from 0 up to 8 (median = 2.36), but only one of the first or second AS in two trials per individual was collected for analysis at each SNR condition. There are the six assumptions for ANOVA: (1) Dependent variable should be continuous; (2) Independent variable consists of two or more categorical, independent groups; (3) Independence of observations, using independent individuals as subjects; (4) There should be no significant outliers; (5) The dependent variable is normally distributed in each group; and (6) Homogeneity of variances. We can check assumptions #4, #5 and #6 using SPSS Statistics. Before doing this, we made sure that our data meets assumptions #1, #2 and #3. Then, we performed a one-way ANOVA test and a Tukey’s honestly significant difference (HSD) test, as a standard post-hoc test, for multiple comparisons using the SPSS Statistics 17.0 software package^45^.

## Additional Information

**How to cite this article**: Shen, J.-X. and Xu, Z.-M. The Lombard effect in male ultrasonic frogs: Regulating antiphonal signal frequency and amplitude in noise. *Sci. Rep.* **6**, 27103; doi: 10.1038/srep27103 (2016).
